# National study on the adequacy of antidotes stocking in Lebanese hospitals providing emergency care

**DOI:** 10.1186/s40360-016-0092-7

**Published:** 2016-11-07

**Authors:** Anthony Mansour, Layla Al-Bizri, Jad El-Maamary, Amanda al-Helou, Rayan Hamade, Elie Saliba, Dina Khammash, Karim Makhoul, Kamal Matli, Nada Ghosn, Mary Deeb, Wissam H. Faour

**Affiliations:** 1 School of Medicine, Lebanese American University, P.O. Box 36, Byblos, Lebanon; 2 Lebanese Ministry of Public Health, Beirut, Lebanon; 3 School of Medicine, Room 4722, Lebanese American University, P.O. Box 36, Byblos, Lebanon

**Keywords:** Antidote stoking, Emergency care, Lebanese hospitals, World health organization

## Abstract

**Background:**

Antidotes stocking is a critical component of hospital care for poisoned patients in emergency. Antidote stocking represents a major health challenge worldwide and in Lebanon. Systematic data monitoring of antidote stocking in Lebanese hospitals is lacking. The objective of this study is to assess the adequacy of antidotes stocking in Lebanese hospitals according to type and quantity and explore the characteristics associated with their differential availability.

**Methods:**

Data collection to assess antidote availability and its correlate was undertaken through a self-administered questionnaire. The questionnaires were distributed by the unit of surveillance at the Ministry of Public Health to eligible hospitals providing emergency care services. The list of essential antidotes was adapted from the World Health Organization (WHO) list and the British Columbia Drug and Poison Information Centre.

**Results:**

Among the 85 Lebanese hospitals surveyed none had in stock all the 35 essential antidotes required. The frequency of stocking by type of antidote varied from a minimum of 1.2 % of the hospitals having a (cyanide kit) to 100 % availability of (atropine and calcium gluconate). Teaching hospitals and those with a large bed-capacity reported a higher number of available antidotes for both immediate and non-immediate use than non-teaching hospitals while controlling for the hospital geographical region and public vs private sector.

**Conclusion:**

The Lebanese hospitals have a suboptimal stock of essential antidotes supply. It is recommended that the Lebanese Ministry of Public Health monitors closely on the hospital premises the adequacy and availability of essential antidotes stock.

**Electronic supplementary material:**

The online version of this article (doi:10.1186/s40360-016-0092-7) contains supplementary material, which is available to authorized users.

## Background

An antidote, as defined by the World Health Organization (WHO), is a therapeutic substance used to counteract the toxic action(s) of a specified xenobiotic [1]. Although emergency supportive care is considered the foundation of toxicological emergency management, unintentional and intentional poisoning continue to be a major contributor to annual mortality rates worldwide [2]. Morbidity, mortality, and the length of hospitalization may be reduced by the appropriate and well-timed use of antidotes [3]. The mainstay in the proper management of a patient in need for an antidote is the immediate administration of the needed antidote and its availability in the hospital emergency. Therefore hospitals and emergency centers should be self-sufficient with regards to antidote stocking. Thus improper stocking or unavailability of the required antidotes are a major factors that lead to mismanagement of poisoned patients.

Data on the availability of antidotes globally is scarce, however recent studies have shown that antidotes are inadequately stocked in many countries and their availability also varies significantly among hospitals within the same country [3–6]. Accordingly, Dart et al., developed recommendations for antidote stocking and categorizing their availability according to the immediacy of their use in USA [7]. However, these recommendations can be only elaborated when information on antidotes stocking in hospitals providing Emergency care is available. Considering such guidelines are still largely missing in Lebanon, it is therefore recommended to first document antidotes stocking before developing the guidelines [7]. Data also shows that antidote stoking varies by the hospital sector (public or private), bed capacity, presence of particular medical specialists (e.g., emergency care), and number of expected poisoning cases [3, 4]. Lebanon, a country of 4 million inhabitants in the Middle East, has a healthcare system dominated by the private sector, where less than 17 % of its hospitals are administered by the public sector [8]. Lebanese hospitals are usually well equipped with state-of-the art medical equipment in the emergency department, however, antidote availability and stocking are not well monitored. The ministry of health reported an increase in number of hospitalized cases due to poisoning by drugs or toxic substances from 288 cases in 2011 to 336 cases in 2012 [8, 9]. A systematic evaluation of the availability of antidote stocking in Lebanese hospitals has not been undertaken to date. The main objective of this study was to document the availability of antidotes considered essential according to the criteria set by the British Columbia Drug and Poison Information Centre (BC DPIC) and the World Health Organization (WHO) [1].

## Methods

### Study design

This is a cross sectional survey study that was undertaken among eligible Lebanese hospitals that have an emergency care setting. The data collection period extended from October 2013 to April 2014 by contacting either the emergency unit or pharmacy department in charge of monitoring and providing antidotes in the hospitals. The Lebanese Ministry of Public Health provided us with a list of addresses and contact information of eligible public and private hospitals with emergency care services in Lebanon. The data collection instrument was a self-administered survey that was distributed by the Lebanese Ministry of Public Health, along with a document explaining the objectives and significance of the study. The questionnaire was filed by the person responsible of stocking the antidotes in the emergency unit or hospital pharmacy. The questionnaire included questions on hospitals geographical distribution, working in the public or private sector and type [university (teaching) vs general (non-teaching)] (Additional file 1: Table S4). Also, the hospitals were stratified according to their bed capacity into small (<50 beds), medium (50–150 beds) and large (>150 beds).

Data on the availability of each antidote from a list of 35 antidotes was also collected as well as the reasons when the antidote was not available (adapted from the British Colombia Drug and Poison Information Centre (BCDPIC) and the World Health Organization) [1]. The latter guidelines were used since there are no current specific guidelines for antidotes stocking in Lebanon.

The list of antidotes were also grouped into “immediate” and “non-immediate” as per WHO guidelines. The group of “immediate” are antidotes given within 30 min to one hour and include: Activated Charcoal; Atropine; Calcium Chloride; Calcium Gluconate; D50W; Digoxin F; Ethanol; Flumanezil; Fomepizole; Glucagon; Glucose; Hydroxycobalamin; Methylene Blue; N-acetylcysteine; Naloxone; Pyridoxine; Na bicarbonate; Na nitrate; Na thiosulfate. The group of “non-immediate” antidotes that can be administered after one hour of poisoning are: Cholestyramine; Deferoxamine; Dimercaprol; EDTA; Folic Acid; Insulin; Isoproterenol; Leucovorin; Magnesium; Octreotide; PEG solution; Pralidoximine; Protamine sulfate; Prostigimine; Vitamin K.

Surveys were distributed by the ministry of Public Health surveillance unit to all eligible hospitals in the first week of October 2013. After two months from the initiation of the study a second reminder was sent and those who did not reply were contacted by telephone. By the end of April 2014 all hospitals that did not reply were labeled as non-respondents.

### Statistical analysis

Data analysis was done using Statistical Package for Social Sciences (SPSS version 23) software for analysis. The distribution of antidotes by categorical data such as type of hospital was performed by using the chi-square test. The independent sample t–test was used to compare the mean antidotes in the different hospital types, sectors, and location. Additionally, separate multiple regression analysis were also performed in order to explore the hospital characteristics as predictors related to two outcomes number of immediate and non-immediate antidote. Results were considered significant with a *p* value of less than 0.05.

## Results

Table 1 shows the characteristics of the 85 sampled hospitals regarding bed capacity, type, and health sector. More than half of the hospitals had a medium (50–150) bed capacity (57.1 %), and the majority were non-teaching (77.4 %) and privately owned (83.3 %) hospitals. Table 2 describes the availability of antidotes according to antidote type, it ranges from 1.2 % having cyanide kit to 100 % having atropine and calcium gluconate. The number of reported antidotes available in each hospital varies from 5–28 with no hospital having the total 35 antidotes in stock (data not shown).

**Table 1:** Characteristics of the hospitals in Lebanon that were evaluated during the study

Characteristic	Percentage
Bed capacity	
• Small (<50 beds)	23.8
• Medium (50–150 beds)	57.1
• Large (> 150 beds)	16.7
Type of hospital	
• Teaching	77.4
• Non-teaching	22.6
Sector	
• Public	16.7
• Private	83.3

**Table 2:** Prevalence of antidote stocking in Lebanese hospitals

Antidotes	Hospitals
	Percent
Atropine	100
Calcium gluconate	100
Glucose	98.8
Insulin	96.4
Sodium bicarbonate	96.4
Magnesium	95.2
Vitamin K	95.2
Naloxone	94.0
Flumazenil	85.7
Protamine sulfate	85.7
NAC	84.5
Prostigmine	83.3
Methylene blue	79.8
Activated charcoal	76.2
Folic acid	67.9
Pralidoxime	63.1
D50W	54.8
Glucagon	52.4
Hydroxycobalamin	44.0
Calcium chloride	44.0
Ethanol	40.5
Octeotride	32.1
PEG solution	41.7
Leucovorin	31.0
Deferoxamine	22.6
Digoxin immune F	21.4
Isoproterenol	20.2
Pyridoxine	19.0
Cholestyramine	6.0
EDTA	6.0
Sodium nitrate	6.0
Fomepizole	3.6
Sodium thiosulfate	3.6
Dimercaprol	2.4
Cyanide Kit	1.2

*PEG* polyethylene glycol electrolyte, *D50W* dextrose 50 % in water, *NAC* N-acetylcysteine, *EDTA* Ethylenediaminetetraacetic acid

Table 3 shows the reasons reported for not having specific antidotes in stock in the hospitals. The major reason was the availability of the antidote in a nearby hospital followed by alternative medications being used instead. Other reported reasons were: unavailable in the Lebanese market, expensive or available in another location within the hospital.

**Table 3:** Reasons reported for Not Stocking Antidotes in Lebanese hospitals

Antidote	Percentage
Available in a nearby hospital (N=13)	
Magnesium	100
Sodium bicarbonate	100
Vitamin K	100
Naloxone	75.0
Protamine sulfate	50.0
NAC	42.8
Fomepizole	37.5
Folic acid	35.7
Glucagon	35.5
Pralidoxime	35.0
Leucovorin	33.3
Hydroxycobalamin	29.2
Ethanol	32.1
Alternative can be used (N=8)	
Activated charcoal	61.5
Calcium chloride	54.2
D50W	52.3
Dimercaprol	44.0
EDTA	40.4
Prostigmine	33.3
PEG solution	30.4
Isoproterenol	30.0
Not available in the market (N=6)	
Cholestyramine	42.3
Cyanide Kit	36.2
Digoxin immune F	43.2
Pyridoxine	39.5
Sodium nitrate	43.8
Sodium thiosulfate	39.2
Expensive (N=3)	
Flumazenil	40.0
Deferoxamine	29.2
Octeotride	25.7
Available in another place within hospital (N=2)	
Insulin	66.6
Methylene blue	37.5

*PEG* polyethylene glycol electrolyte, *D50W* dextrose 50 % in water, *NAC* N-acetylcysteine, *EDTA* Ethylenediaminetetraacetic acid

### Distribution of antidotes by hospital geographical location

The hospitals located in Beirut and its suburbs have a slightly higher number of antidotes in stock with a mean of 19.05 ± 5.27 compared to 18.15 ± 4.04 in the other governorates (*p* = 0.39). The mean number of antidote availability for immediate and non-immediate by geographic location was also not significant (Table 4). Of note, there were significant differences between the region of the hospital and only 5 different antidotes (Additional file 2: Table S1).

**Table 4:** Analysis of immediate and non-immediate antidotes availability according to hospital type, geographic area and hospital sector

		N	Mean	Std. deviation	Std. error mean	*P*-value
Mean number of immediate and non-immediate antidotes availability according to hospital type						
Immediate	Non-teaching	65	10.6308	2.69588	0.33438	0.001
	Teaching	19	12.4737	1.71167	0.39268	
Non-immediate	Non-teaching	65	6.8308	2.13285	0.26455	0
	Teaching	19	9.7368	1.75885	0.40351	
Analysis of antidote availability according to geographic area (region)						
Immediate	Greater Beirut	37	11.1622	2.88207	0.47381	0.315
	Others	47	10.9574	2.41335	0.35202	
Non-immediate	Greater Beirut	37	7.8919	2.70579	0.44483	0.04
	Others	47	7.1702	2.06755	0.30158	
Mean number of antidote availability according to hospital sector						
Immediate	Private	70	11.1571	2.71141	0.32408	0.394
	Public	14	10.5	2.06621	0.55222	
Non-immediate	Private	70	7.6714	2.48311	0.29679	0.04
	Public	14	6.5714	1.55486	0.41555	

### Distribution of antidote by type of hospital

#### Private vs public

The distribution of the antidotes by hospital health sector shows that the mean availability of antidote was 18.84 ± 4.85 in private hospitals versus 17.07 ± 2.79 in public hospitals (*p* = 0.07), but a significant difference was found between the availability of seven antidotes (Digoxin immune F, Fomepizole, Glucagon, Octeotride, Leucovorin, Isoproterenol, Pyridoxine) and the hospital sector. Furthermore, atropine, calcium gluconate and flumazenil were equally available regardless the hospital sector (Additional file 3: Table S2). The mean number of immediate antidotes available by type of hospital (Private vs Public) did not differ significantly (*p*-value = 0.315). However, the mean number of 7.7 for non-immediate antidote availability in Private hospitals compared to 6.5 among Public hospitals was significantly different with a *p*-value = 0.04 (Table 4).

### Teaching vs non-Teaching

Additionally, our data showed that teaching hospitals stocked an average of 22.21 ± 3.26 antidotes compared to 17.48 ± 4.41 in non-teaching hospitals (*p* < 0.0001) and there was a significant difference between the hospital type and nine different antidotes (Additional file 4: Table S3). The mean number of immediate and non-immediate antidote by type of hospital also differed significantly. The mean number in non-teaching hospital for immediate was 10.6 compared to 12.5 among Teaching hospitals. For non-immediate Non-Teaching 6.8 and Teaching 9.7 (Table 4).

### Bed capacity

**Fig. 1:**
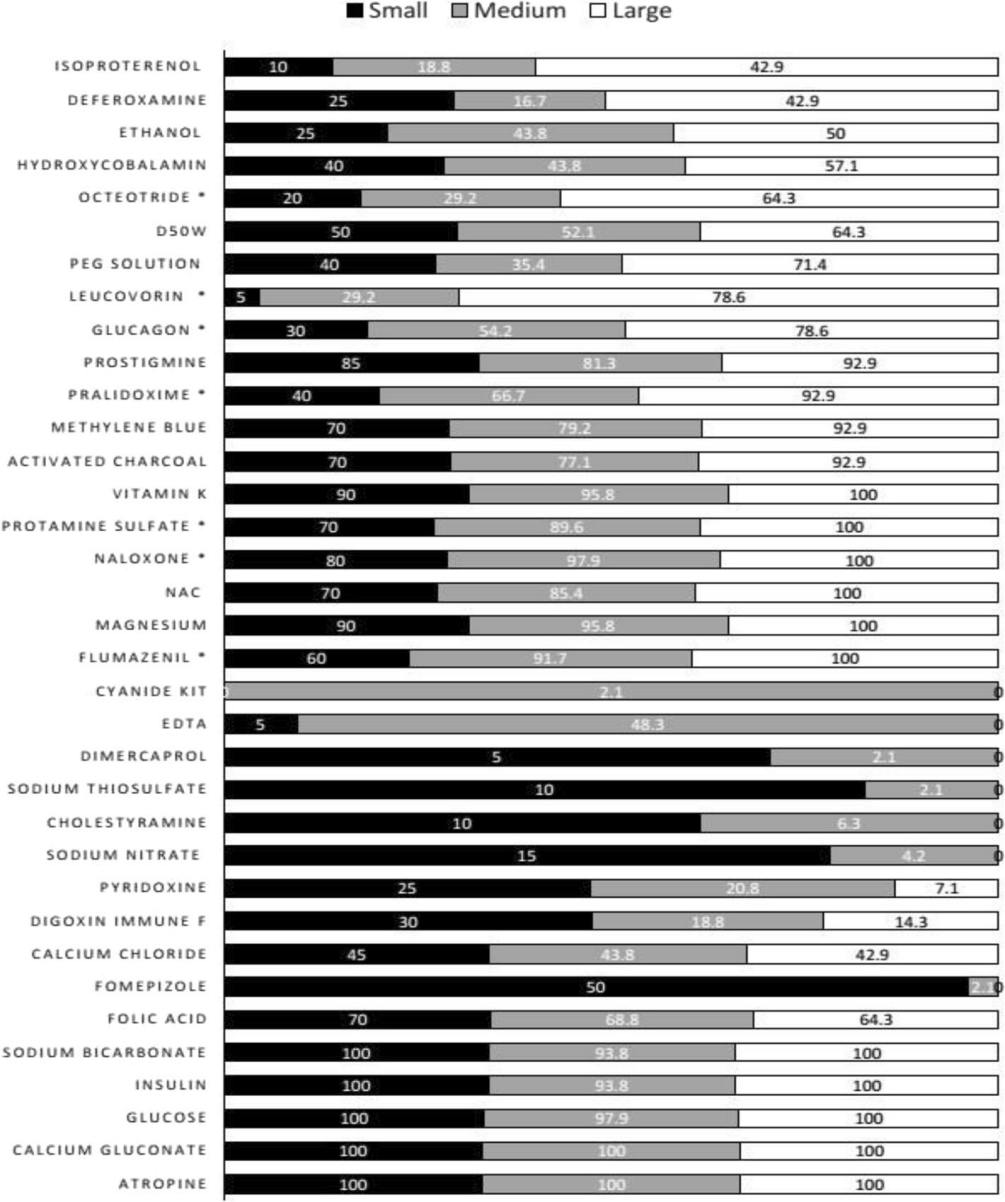
Represents the distribution of the different antidotes among small (24.40 %), medium (58.50 %) and large (17.10 %) hospitals. ^*^ *p* < 0.05 significance different between hospital sizes and each specific antidote

According to the bed capacity the mean availability was 16.90 ± 4.64, 18.48 ± 4.59, and 21.50 ± 3.46 for small, medium and large hospitals respectively (*p* = 0.014), and a significant difference was found between the hospital size and the availability of seven different antidotes (octeotride, leucovorin, glucagon, pralidoxime, protamine sulfate, naloxone, flumazenil). Moreover, glucose, insulin, atropine, calcium gluconate and sodium bicarbonate were equally available in small and large hospitals (Fig. 1).

Multiple regression analysis showed that the main predictor of the number of antidote availability is being a teaching compared to non-teaching hospitals (*P*-value = 0.01) while controlling for hospital sector, geographical area and bed capacity. Multiple regression analysis were also done for immediate and non-immediate antidote separately. Finally, multiple regression analysis showed a similar result as observed for the total number of antidotes where teaching hospitals again seem to have the higher probability of having available both immediate and non-immediate significantly more than non-teaching hospitals (Tables 5, 6 and 7).

**Table 5:** Regression analysis of the mean antidote availability according to hospital type (teaching vs non-teaching), geographical region and bed capacity

Coefficients^a^						
Model		Unstandardized coefficients		Standardized coefficients	t	Sig.
		B	Std. error	Beta		
1	(Constant)	11.239	3.059		3.674	.000
	Hospital type	4.674	1.315	.429	3.554	.001
	Sector	-1.780	1.389	-.137	-1.281	.204
	Region	1.294	1.038	.140	1.247	.216
	Bed capacity	.856	.848	.119	1.010	.316

^a^Dependent Variable: Mean antidotes

**Table 6:** Regression analysis of the mean “immediate” antidote availability according to hospital type (teaching vs non-teaching), sector, geographical region and bed capacity region

Coefficients^a^						
Model		Unstandardized coefficients		Standardized coefficients	t	Sig.
		B	Std. error	Beta		
1	(Constant)	8.116	1.845		4.399	.000
	Hospital type	1.970	.793	.319	2.483	.015
	Sector	-.970	.838	-.132	-1.158	.250
	Region	.670	.626	.128	1.070	.288
	Bed capacity	.295	.511	.073	.578	.565

^a^Dependent Variable: Immediate

**Table 7:** Regression analysis of the mean “not- immediate” antidote availability according to hospital type (teaching vs non-teaching), sector, geographical region and bed capacity region

Coefficients^a^						
Model		Unstandardized coefficients		Standardized coefficients	t	Sig.
		B	Std. error	Beta		
1	(Constant)	3.133	1.486		2.109	.038
	Hospital type	2.715	.639	.488	4.250	.000
	Sector	-.791	.675	-.119	-1.171	.245
	Region	.599	.504	.127	1.188	.239
	Bed capacity	.552	.412	.150	1.339	.184

^a^Dependent Variable: non-immediate

## Discussion

The main objective of this study was to evaluate the adequacy of antidote stocking in Lebanese hospitals providing emergency care services. Our results showed that most hospitals had an insufficient number of the main required antidotes which is consistent with other studies from multiple countries [4, 10–12]. Hospitals with a larger bed capacity had stocked more antidotes when compared to smaller hospitals and these results were similar to data found by others [6, 10, 12–14]. Moreover, we found that the mean number of antidotes that are used either both immediately or non-immediately is significantly different between types of hospitals with teaching hospitals stocked higher number in each category. Such finding is extremely important knowing that most of the teaching hospitals are located in the capital area while hospitals located away from the hospital might lack lifesaving antidotes. The negative impact of lacking important antidotes in areas far from the capital can be minimize since the geographic area of Lebanon is small enough rendering the communication between hospitals an achievable task. In conclusion, antidote stoking of the immediate category needs to be reevaluated particularly in hospitals located outside the capital in order to prevent and reduce the cost of care associated with the lack of such antidotes. Regarding poisoning cases requiring the use of non-immediate antidotes, the health impact of lacking of such antidotes in a particular hospital can be minimized since major hospitals can be reached at reasonable time before irreversible organ damage occurs since the geographic area of Lebanon is relatively small.

Several reasons for not stocking antidotes in smaller hospitals were reported in a recent study in Australia such as budget constraints, perceived lack of antidote need, short expiry dates or the rapid transfer of patients or antidotes [6]. Moreover, inadequate stocking of antidotes in hospitals has also been attributed to the short shelf-life, cost, and lack of awareness about the risks of intoxication. Additionally, hospitals may waive the stocking of antidotes that can be quickly obtained from neighboring institutions in case of an emergency. Finally, one of the key element in the inadequacy of antidotes stocking in the hospital pharmacy is the absence of stocking and management guidelines as well as their implementation [11]. Worthy to note that the number of antidotes among hospitals did significantly differ according to the geographic area emphasizing the role of the LMPH to provide hospitals in far areas with enough medical resources. However, better policies of antidotes stockings are needed to further uncover the needs for particular antidotes in these hospitals. For instance, atropine is available in all hospitals located nearby agriculture areas (usually far from the capital) where the risk of exposure to pesticide poisoning (organophosphates and carbamate poisoning) is high. Of note, pesticide poisoning is becoming a major problem globally and causing thousands of death, it necessitate immediate infusion of the antidote “atropine” along with ventilation support [15, 16]. Hence, the availability of atropine in these hospitals is critical and its availability can limit the catastrophic outcomes associated with delaying treatment.

Cyanide poisoning requires immediate and aggressive treatment and its clinical diagnosis is usually difficult as no specific symptoms are present, whereas laboratory findings require hours to confirm a diagnosis [17]. Therefore, an antidote should be administered when cyanide poisoning is suspected. In Lebanon, the cyanide kit is the least available antidote which raises concern when a case of cyanide poisoning is suspected. Other alternatives approved for empirical therapy in cyanide poisoning include sodium thiosulfate and hydroxycobalamin [18], but they are available in less than 50 % of all hospitals, hence cases of cyanide poisoning cannot be adequately treated. Even though in such incidences the antidotes can be requested from nearby hospitals, the issue of delivery time remains the main concern in Lebanon due to traffic jams or distances between hospitals.

On the other hand, no association has been documented between the hospital sector and the mean availability of antidotes. Subsequently, fomepizole and digoxin immune F, two expensive antidotes used in the treatment of alcohol and digoxin toxicity respectively, were significantly present in public rather than private hospitals highlighting the fact that the public sector funded by the ministry of health had better capabilities of obtaining more expensive antidotes. The number of public hospitals in Lebanon account for 17 % of the total hospitals [8] and the difference of antidote stocking can be attributed to the different poisoning cases encountered and the budget drafted by each of the hospitals sectors. Although, Greater Beirut area has a greater population and more poisoning cases would be expected and managed in this area as compared to the other governorates, an equal distribution of the number of antidotes was documented among all regions in Lebanon and thus there was no centralization among the distribution of antidotes in the capital area and its suburbs. On the other hand, the fact that university hospitals acquired a larger number of antidotes when compared to non-teaching hospitals can be attributed to the higher funding of university hospitals and the variety of clinical exposures needed to be provided to medical students.

Although, this study was relied on antidote lists developed both locally and externally, which may not accurately reflect the coverage required for toxicological emergencies in Lebanon, it may represent an important step toward implementing better antidote stocking policies. Since hospitals didn’t often report the quantity of each antidote they possess so we were unable to determine whether an antidote was stocked in sufficient amount.

## Conclusions

This study showed an inadequate antidote stocking in the Lebanese hospitals and that the mean number of antidote available was significantly greater in teaching hospitals vs non-teaching hospitals. Establishing a national antidote database to enhance communication between health institutions regarding their stocks of antidote will lead to a better and effective antidote stocking system. The Lebanese Ministry of Public Health should also review and implement new guidelines regarding stocking of antidotes, taking into consideration the WHO recommendations and the type of toxicological cases in Lebanon.

## Additional files

Additional file 1: Table S4. Questionnaire. (DOCX 17 kb)
Additional file 2: Table S1. Variation in the Distribution of Antidotes between Greater Beirut and Other Governorates. (DOC 74 kb)
Additional file 3: Table S2. Distribution of Antidotes by Sector type; private –public. (DOC 65 kb)
Additional file 4: Table S3. Distribution of Antidotes by type of hospitals. (DOC 54 kb)

## Abbreviations

BCDPIC: British Colombia drug and poison information centre
LMPH: Lebanese ministry of public health
WHO: World health organization

## References

[1] International Program on Chemical Safety., World Health Organization (1997). Guidelines for poison control.

[2] Watson WA, Litovitz TL, Klein-Schwartz W, Rodgers GC, Youniss J, Reid N, Rouse WG, Rembert RS, Borys D (2004). 2003 annual report of the American Association of Poison Control Centers Toxic Exposure Surveillance System. Am J Emerg Med.

[3] Al-Sohaim SI, Awang R, Zyoud SH, Rashid SM, Hashim S (2012). Evaluate the impact of hospital types on the availability of antidotes for the management of acute toxic exposures and poisonings in Malaysia. Hum Exp Toxicol.

[4] Gasco L, Rosbolt MB, Bebarta VS (2013). Insufficient stocking of cyanide antidotes in US hospitals that provide emergency care. J Pharmacol Pharmacother.

[5] Juurlink DN, McGuigan MA, Paton TW, Redelmeier DA (2001). Availability of antidotes at acute care hospitals in Ontario. CMAJ.

[6] Nissen LM, Wong KH, Jones A, Roberts DM (2010). Availability of antidotes for the treatment of acute poisoning in Queensland public hospitals. Aust J Rural Health.

[7] Dart RC, Borron SW, Caravati EM, Cobaugh DJ, Curry SC, Falk JL, Goldfrank L, Gorman SE, Groft S, Heard K (2009). Expert consensus guidelines for stocking of antidotes in hospitals that provide emergency care. Ann Emerg Med.

[8] Department of statistics TLMoPH (2012). Statistical Bulletin.

[9] Department of Statistics MoPH (2011). Statistical Bulletin.

[10] Dart RC, Stark Y, Fulton B, Koziol-McLain J, Lowenstein SR (1996). Insufficient stocking of poisoning antidotes in hospital pharmacies. JAMA.

[11] Jean-François Bussières BB. Insufficient Stocking of Antidotes in Hospital Pharmacies: Problem, Causes, and Solution. Can J Hosp Pharm. 2000;53.

[12] Ong HC, Yang CC, Deng JF (2000). Inadequate stocking of antidotes in Taiwan: is it a serious problem?. J Toxicol Clin Toxicol.

[13] Abbott V, Creighton M, Hannam J, Vincent T, Coulter C (2012). Access in New Zealand to antidotes for accidental and intentional drug poisonings. J Prim Health Care.

[14] Higgins MA, Evans R (2000). Antidotes--inappropriate timely availability. Hum Exp Toxicol.

[15] Eddleston M, Phillips MR (2004). Self poisoning with pesticides. BMJ.

[16] Heath AJW MT (1992). Atropine in the management of anticholinesterase poisoning. Clinical and experimental toxicology of organophosphates and carbamates.

[17] Hall AH, Rumack BH (1986). Clinical toxicology of cyanide. Ann Emerg Med.

[18] Sauer SW, Keim ME (2001). Hydroxocobalamin: improved public health readiness for cyanide disasters. Ann Emerg Med.

