# Supplementary material for: National study on the adequacy of antidotes stocking in Lebanese hospitals providing emergency care
Source: BMC Pharmacol Toxicol. 2016 Nov 7;17:51. doi: 10.1186/s40360-016-0092-7 (PMC5098286; doi:10.1186/s40360-016-0092-7)
Supplement: Additional file 1: Table S4. — Questionnaire. (DOCX 17 kb) [file 40360_2016_92_MOESM1_ESM.docx]

***General Information:***

1. Hospital Name: ______________________________________ **2.** Hospital Type: General □ University □
2. Hospital Address: Village: __________________­­__ Qadaa/Kaza:_______________________­­­­­­­­____

**4.** Sector: Private □ Public □ **5.** Number of Operational Beds: ____________

**6.** Emergency Care Center: Yes (24/7) □ Yes (not 24/7) □ No □

***Toxicological Information:***

1. Incidence of chemical toxicological poisoning cases in the last 2 months: Suspected ­­­­_______ Confirmed:_______
2. Complete the following table and place a check mark in the appropriate columns.

“Reason for not stocking”: **A**. Expensive **B**. Short shelf life **C**. Available in nearby hospital **D**. Alternative can be used

| **Name of Antidote** | **present** | | | **Stocking Capacity of the Antidote (grams, if other specific)** | **Reason for Not Stocking (use above choices)** | | | | |
| --- | --- | --- | --- | --- | --- | --- | --- | --- | --- |
|  | **yes** | **No** |  | | **A** | **B** | **C** | **D** | **Others** |
| **Activated Charcoal** |  |  |  | |  |  |  |  |  |
| **Atropine** |  |  |  | |  |  |  |  |  |
| **Calcium Chloride** |  |  |  | |  |  |  |  |  |
| **Calcium Gluconate** |  |  |  | |  |  |  |  |  |
| **Cholestyramine** |  |  |  | |  |  |  |  |  |
| **Cyanide Kit** |  |  |  | |  |  |  |  |  |
| **D_50_W** |  |  |  | |  |  |  |  |  |
| **Deferoxamine** |  |  |  | |  |  |  |  |  |
| **Digoxin Immune F** |  |  |  | |  |  |  |  |  |
| **Dimercaprol** |  |  |  | |  |  |  |  |  |
| **EDTA** |  |  |  | |  |  |  |  |  |
| **Ethyl Alcohol (ethanol)** |  |  |  | |  |  |  |  |  |
| **Flumazenil** |  |  |  | |  |  |  |  |  |
| **Folic Acid** |  |  |  | |  |  |  |  |  |
| **Fomepizole** |  |  |  | |  |  |  |  |  |
| **Glucagon** |  |  |  | |  |  |  |  |  |
| **Glucose** |  |  |  | |  |  |  |  |  |
| **Hydroxycobalamin** |  |  |  | |  |  |  |  |  |
| **Insulin** |  |  |  | |  |  |  |  |  |
| **Isoproterenol** |  |  |  | |  |  |  |  |  |
| **Leucovorin** |  |  |  | |  |  |  |  |  |
| **Magnesium** |  |  |  | |  |  |  |  |  |
| **Methylene Blue** |  |  |  | |  |  |  |  |  |
| **N-Acetylcysteine (NAC)** |  |  |  | |  |  |  |  |  |
| **Naloxone** |  |  |  | |  |  |  |  |  |
| **Octreotride** |  |  |  | |  |  |  |  |  |
| **PEG Solution** |  |  |  | |  |  |  |  |  |
| **Physostigmine/Prostigmine** |  |  |  | |  |  |  |  |  |
| **Pralidoxime** |  |  |  | |  |  |  |  |  |
| **Protamine Sulfate** |  |  |  | |  |  |  |  |  |
| **Pyridoxine** |  |  |  | |  |  |  |  |  |
| **Sodium Bicarbonate** |  |  |  | |  |  |  |  |  |
| **Sodium Nitrite** |  |  |  | |  |  |  |  |  |
| **Sodium Thiosulfate** |  |  |  | |  |  |  |  |  |
| **Vitamin K** |  |  |  | |  |  |  |  |  |
