# Supplementary material for: National study on the adequacy of antidotes stocking in Lebanese hospitals providing emergency care
Source: BMC Pharmacol Toxicol. 2016 Nov 7;17:51. doi: 10.1186/s40360-016-0092-7 (PMC5098286; doi:10.1186/s40360-016-0092-7)
Supplement: Additional file 2: Table S1. — Variation in the Distribution of Antidotes between Greater Beirut and Other Governorates. (DOC 74 kb) [file 40360_2016_92_MOESM2_ESM.doc]

|  |  | **% of Available Antidote** | |
| --- | --- | --- | --- |
| **Greater Beirut vs. Other Governorates** | **Antidotes** | **Greater Beirut** | **Other Governorates** |
| **Same (N=2)** | Atropine | 100 | 100 |
|  | Calcium gluconate | 100 | 100 |
| **Greater (N=18)** | Glucagon | 100 | 97.9 |
|  | Magnesium | 97.3 | 93.6 |
|  | Naloxone | 97.3 | 91.5 |
|  | Vitamin K | 97.3 | 93.6 |
|  | Flumazenil | 91.9 | 80.9 |
|  | Protamine sulfate | 89.2 | 83 |
|  | Prostigmine | 86.5 | 80.9 |
|  | Activated charcoal | 83.8 | 70.2 |
|  | Methylene blue | 83.6 | 76.6 |
|  | Pralidoxime | 70.3 | 57.4 |
|  | Glucose | 59.5 | 46.8 |
|  | PEG solution* | 54.1 | 31.9 |
|  | Leucovorin* | 45.9 | 19.1 |
|  | Octreotide* | 45.9 | 17.3 |
|  | Ethanol | 45.9 | 36.2 |
|  | Deferoxamine | 24.3 | 21.3 |
|  | Digoxin immune F | 24.3 | 19.1 |
|  | Isoproterenol | 27 | 14.9 |
| **Less (N=15)** | Insulin | 94.6 | 97.9 |
|  | Sodium bicarbonate | 94.6 | 97.9 |
|  | NAC | 83.8 | 85.1 |
|  | Folic acid* | 51.4 | 80.9 |
|  | D_50_W | 48.6 | 59.6 |
|  | Calcium chloride | 40.5 | 46.8 |
|  | Hydroxycobalamin | 40.5 | 46.8 |
|  | Pyridoxine | 18.9 | 19.1 |
|  | Sodium nitrate* | 0 | 10.6 |
|  | Cholestyramine | 2.7 | 8.5 |
|  | EDTA | 2.7 | 8.5 |
|  | Fomepizole | 0 | 6.4 |
|  | Dimercaprol | 0 | 4.3 |
|  | Sodium thiosulfate | 2.7 | 4.3 |
|  | Cyanide Kit | 0 | 2.1 |
|  | **Mean ± SD** | 19.05 ± 5.27 | 18.15 ± 4.04 |
| Peripheral Governorates (57.0%) Capital and Suburbs (43.0%)  PEG: polyethylene glycol electrolyte, D_50_W: dextrose 50% in water, NAC: N-acetylcysteine, EDTA: Ethylenediaminetetraacetic acid.  *p < 0.05 significance difference between greater Beirut and other governorates for each specific antidote. | | | |

**Additional file 2: Table S1. Variation in the Distribution of Antidotes between Greater Beirut and Other Governorates**
