# Supplementary material for: National study on the adequacy of antidotes stocking in Lebanese hospitals providing emergency care
Source: BMC Pharmacol Toxicol. 2016 Nov 7;17:51. doi: 10.1186/s40360-016-0092-7 (PMC5098286; doi:10.1186/s40360-016-0092-7)
Supplement: Additional file 3: Table S2. — Distribution of Antidotes by Sector type; private –public. (DOC 65 kb) [file 40360_2016_92_MOESM3_ESM.doc]

**Additional file 3: Table S2. Distribution of Antidotes by Sector type: private – Public**

|  |  | **% of Available antidote** | |
| --- | --- | --- | --- |
| **Private vs Public Hospitals** | **Antidotes** | **Private** | **Public** |
| **Same (N=3)** | Atropine | 100 | 100 |
|  | Calcium gluconate | 100 | 100 |
|  | Flumazenil | 85.7 | 85.7 |
| **Less (N=13)** | Glucose | 98.6 | 100 |
|  | Insulin | 95.7 | 100 |
|  | Naloxone | 92.9 | 100 |
|  | Sodium bicarbonate | 95.7 | 100 |
|  | Vitamin K | 94.3 | 100 |
|  | Prostigmine | 82.9 | 85.7 |
|  | Folic acid | 67.1 | 71.4 |
|  | Pralidoxime | 62.9 | 64.3 |
|  | D_50_W | 54.3 | 57.1 |
|  | Digoxin immune F * | 7.1 | 42.9 |
|  | Fomepizole * | 1.4 | 14.3 |
|  | Cholestyramine | 5.7 | 7.1 |
|  | Sodium nitrate | 5.7 | 7.1 |
| **Greater (N=18)** | Magnesium | 95.7 | 92.9 |
|  | NAC | 85.7 | 78.6 |
|  | Methylene blue | 81.4 | 71.4 |
|  | Protamine sulfate | 81.4 | 78.6 |
|  | Activated charcoal | 77.1 | 71.4 |
|  | Glucagon * | 58.6 | 21.4 |
|  | Hydroxycobalamin | 47.1 | 28.6 |
|  | PEG solution | 45.7 | 21.4 |
|  | Calcium chloride | 44.3 | 42.9 |
|  | Ethanol | 42.9 | 28.6 |
|  | Octreotide * | 37.1 | 7.1 |
|  | Leucovorin * | 35.7 | 7.1 |
|  | Isoproterenol * | 24.3 | 0 |
|  | Deferoxamine | 22.9 | 21.4 |
|  | Pyridoxine * | 22.9 | 0 |
|  | EDTA | 7.1 | 0 |
|  | Sodium thiosulfate | 4.3 | 0 |
|  | Dimercaprol | 2.9 | 0 |
|  | **Mean ± SD** | **18.84 ± 4.85** | **17.07 ± 2.79** |
| **Private Hospitals (83.3%); Publics Hospitals (16.7%)**  PEG: polyethylene glycol electrolyte, D_50_W: dextrose 50% in water, NAC: N-acetylcysteine, EDTA: Ethylenediaminetetraacetic acid.  *p < 0.05 | | | |
