# Supplementary material for: National study on the adequacy of antidotes stocking in Lebanese hospitals providing emergency care
Source: BMC Pharmacol Toxicol. 2016 Nov 7;17:51. doi: 10.1186/s40360-016-0092-7 (PMC5098286; doi:10.1186/s40360-016-0092-7)
Supplement: Additional file 4: Table S3. — Distribution of Antidotes by type of hospitals. (DOC 54 kb) [file 40360_2016_92_MOESM4_ESM.doc]

**Additional file 4: Table S3. Distribution of Antidotes by type of hospital**

|  |  | **% of Available Antidotes** | |
| --- | --- | --- | --- |
| **Teaching vs. Non-teaching** | **Antidotes** | **Non-Teaching** | **Teaching** |
| **Same (N=2)** | Atropine | 100 | 100 |
|  | Calcium gluconate | 100 | 100 |
| **Greater (N=9)** | Calcium chloride | 44.6 | 42.1 |
|  | Pyridoxine | 20 | 15.8 |
|  | EDTA | 7.7 | 0 |
|  | Sodium nitrate | 7.7 | 0 |
|  | Cholestyramine | 6.2 | 5.3 |
|  | Fomepizole | 4.6 | 0 |
|  | Sodium thiosulfate | 4.6 | 0 |
|  | Dimercaprol | 3.1 | 0 |
|  | Cyanide Kit | 1.5 | 0 |
| **Less (N=24)** | Flumazenil * | 81.5 | 100 |
|  | Glucose | 98.5 | 100 |
|  | Insulin | 95.4 | 100 |
|  | Magnesium | 93.8 | 100 |
|  | Naloxone | 92.3 | 100 |
|  | Sodium bicarbonate | 95.4 | 100 |
|  | Protamine sulfate * | 81.5 | 100 |
|  | Vitamin K | 93.8 | 100 |
|  | Methylene blue | 75.4 | 94.7 |
|  | NAC | 81.5 | 94.7 |
|  | Prostigmine | 80 | 94.7 |
|  | Activated charcoal | 72.3 | 89.5 |
|  | Pralidoxime * | 55.4 | 89.5 |
|  | Glucagon * | 43.1 | 84.2 |
|  | Folic acid | 64.6 | 78.9 |
|  | Leucovorin * | 16.9 | 78.9 |
|  | Octreotide * | 18.5 | 78.9 |
|  | D_50_W | 49.2 | 73.7 |
|  | PEG solution * | 32.3 | 73.7 |
|  | Ethanol * | 32.3 | 68.4 |
|  | Hydroxycobalamin | 40 | 57.9 |
|  | Deferoxamine | 18.5 | 36.8 |
|  | Isoproterenol * | 15.4 | 36.8 |
|  | Digoxin immune F | 20 | 26.3 |
|  | **Mean ± SD** | **17.48 ± 4.41** | **22.21 ± 3.26^#^** |
| Independent hospitals (77.4%) University affiliated hospitals (22.6%).  PEG: polyethylene glycol electrolyte, D_50_W: dextrose 50% in water, NAC: N-acetylcysteine, EDTA: Ethylenediaminetetraacetic acid.  *p < 0.05 significant difference between independent and university affiliated hospital for each antidote.  ^#^p < 0.0001 | | | |
